# The complete mitochondrial genome of a vulnerable mandarin fish *Coreoperca liui* (Teleostei: Perciformes: Serranidae) from Qiandaohu Lake in China

**DOI:** 10.1080/23802359.2022.2086081

**Published:** 2022-06-30

**Authors:** Fangyuan Guan, Qiang Sheng, Yixiang Zhang, He Lv, Yingying Wang

**Affiliations:** a College of Life Sciences, Zhejiang Province Key Laboratory of Aquatic Resources Conservation and Development, Huzhou University, Huzhou, China; b National Wetland Museum of China, Hangzhou, China

**Keywords:** *Coreoperca liui*, Sinipercidae, complete mitochondrial genome, freshwater fish, phylogenetic analysis

## Abstract

*Coreoperca liui* as an approximate species of the genus *Siniperca*, provides an important source for the genetic diversity of the mandarin fish, which is valuable for the protection of biodiversity and utilization of germplasm resources. The complete mitochondrial genome of *C. liui* is 16,482 bp long and it consists of 13 protein-coding genes (PCGs), two ribosomal RNA genes, 22 transfer RNA genes, and a control region (D-loop). Phylogenetic analysis using the maximum-likelihood method, based on 13 PCGs and two rRNA from 13 species produced three major clades. The phylogenetic tree showed that *C. liui* is most closely related to *Coreoperca whiteheadi*. Our results provide useful information for understanding the phylogeny of the genus *Coreoperc*a, as well as for conducing conservation studies of Sinipercidae and related species.

*Coreoperca liui* (Cao and Liang 2013) is a member of the order Perciformes and it belongs to the family Sinipercidae, one of the most diverse groups of mandarin fish. They usually live in streams with rapid water flow and good water quality (Cao and Liang 2013). The distribution of *C. liui* is in the southeast coastal areas of China, where the species is mainly distributed in the lower reaches of the Yangtze River, Pearl River, and other river basins (Li 1991; Song et al. 2017). Its habitat is rapidly deteriorating owing to industrial wastewater discharge and hydropower dam construction (Lin et al. 2019; Liu et al. 2019). There were only few reports on the genetic research of *C. liui*, and no report on its complete mitogenome. The analysis of mitochondrial DNA fragment and complete mitogenome have been successfully applied in fish identification, phylogenetic analysis, and population biology (Billington and Hebert 1991).

In this study, the complete mitogenome of *C. liui* was determined through Illumina Hiseq sequencing (GenBank accession number: MZ964309). The fish was sampled from Qiandaohu Lake, located in Zhejiang Province, China (29.37°N, 118.73°E). The sample was preserved in 95% ethanol and deposited at Huzhou University (www.zjhu.edu.cn, Yixiang Zhang, yxzhang@zjhu.edu.cn) under the voucher number HZ202010211. The total genomic DNA was extracted from fish muscles following the method described in Tang et al. (2008), and then sequenced using Illumina HiSeq4000 (Han et al. 2020). After sequencing, the complete mitogenome was assembled through NOVOPlasty (https://github.com/ndierckx/NOVOPlasty), and annotated using MITOS (http://mitos2.bioinf.uni-leipzig.de/index.py) (Dierckxsens et al. 2016; Donath et al. 2019). It was also annotated using *Coreoperca whiteheadi* (KJ149811.1 in GenBank) as a reference (Lv et al. 2016).

The entire mitochondrial genome of *C. liui* is a circular molecule with a length of 16,482 bp, which consists of 13 protein-coding genes (PCGs), two ribosomal RNA (rRNA) genes, 22 transport RNA (tRNA) genes, and a control region (D-loop). The overall nucleotide composition is 28% A, 26.69% T, 29.24% C, and 16.07% G. The content of A + T is 54.69%, which shows an obvious AT preference, and the gene content and arrangement are similar to the mitochondrial genome of typical vertebrates. *ND6* and eight tRNAs (*tRNA^Gln^*, *RNA^Ala^*, *tRNA^Asn^*, *tRNA^Cys^*, *tRNA^Tyr^*, *tRNA^Ser^*, *tRNA^Glu^*, *tRNA^Pro^*) are encoded on the L-strand, while the others are encoded on the H-strand. Two types of start codons (ATG, GTG) and four types of stop codons (TAG, TAA, TA–, T––) were used in the 13 PCGs. Most of the PCGs start with ATG, while the codon of *COI* is GTG. Six PCGs were terminated with the complete stop codons TAA or TAG; *ATPase6* and *COIII* were terminated with incomplete codon (TA–); and five protein coding genes (*ND2*, *ND3*, *ND4*, *Cytb*, *COII*) were terminated with the incomplete codon (T––), which was similar to past reports on the mitochondrial genes of other fishes. The truncated stop codons TA– and T–– are very common in animals, which are presumably completed as TAA by post-transcriptional polyadenylation (Boore 1999).

Comparisons between *C. liui* and 12 other species using the *COI* gene and 13 PCGs from NCBI (https://www.ncbi.nlm.nih.gov/nuccore/MZ964309/) showed that the sequence identity between *C. liui* and *C. whiteheadi* is the highest at approximately 87.96%, and the sequence identities with *C. herzi*, *C. kawamebari*, and *Siniperca scherzeri* are 87.69%, 86.07%, and 83.46%, respectively (Yamanoue et al. 2007; Chu et al. 2013; Park et al. 2016). The molecular phylogenetic tree was constructed based on two rRNA and 13 protein coding genes from *C. liui* and 12 others related species of the subfamily Serranidae with the species *Aethaloperca rogaa* (Forsskål, 1775) as an outgroup, using the maximum-likelihood method with 1000 replicates in IQ-tree 2.1.2 (http://www.iqtree.org/). The most suitable nucleotide substitution pattern (TPM2 + F + R3) was selected on the basis of the BIC (Minh et al. 2020) (Figure 1). According to our results, *C. liui* had a closer relationship with *C. whiteheadi* than with the other four species of *Coreoperca*, in agreement with the result of the *COI*-based BLAST analysis in NCBI. This mitochondrial genome provides important genomic information on the genus *Coreoperca* that may contribute to biodiversity protection and phylogenetic analysis of Serranidae.

**Figure 1.**
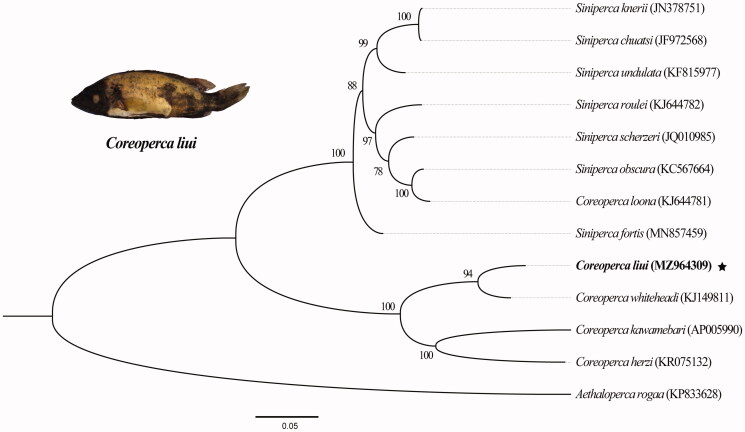
The maximum-likelihood tree of *Coreoperca liui* and 12 other species based on the combined sequences of 13 protein-coding genes and two ribosomal RNA genes. Bootstrap confidences intervals are shown at the nodes. ^★^The newly sequenced mitogenome.

## Data Availability

The genome sequence data that support the findings of this study are openly available in GenBank of NCBI at https://www.ncbi.nlm.nih.gov/, under the accession no. MZ964309. The associated BioProject, Bio-Sample, and SRA accession numbers are PRJNA825346, SAMN27512414, and SRR18709848, respectively.
